# House dust mite major allergens Der p 1 and Der p 5 activate human airway-derived epithelial cells by protease-dependent and protease-independent mechanisms

**DOI:** 10.1186/1476-7961-4-5

**Published:** 2006-03-28

**Authors:** Henk F Kauffman, Michael Tamm, J André B Timmerman, Peter Borger

**Affiliations:** 1 Department of Allergology, University Medical Centre Groningen, Hanzeplein 1, Groningen, The Netherlands; 2 Pulmonary Cell Research, University Hospital Basel, Hebelstrasse 20, Basel, Switzerland

## Abstract

House dust mite allergens (HDM) cause bronchoconstriction in asthma patients and induce an inflammatory response in the lungs due to the release of cytokines, chemokines and additional mediators. The mechanism how HDM components achieve this is largely unknown. The objective of this study was to assess whether HDM components of *Dermatophagoides pteronissinus *with protease activity (Der p 1) and unknown enzymatic activity (Der p 2, Der p 5) induce biological responses in a human airway-derived epithelial cell line (A549), and if so, to elucidate the underlying mechanism(s) of action. A549 cells were incubated with HDM extract, Der p 1, recombinant Der p 2 and recombinant Der p 5. Cell desquamation was assessed by microscopy. The proinflammatory cytokines, IL-6 and IL-8, were measured by ELISA. Intracellular Ca^2+ ^levels were assessed in A549 cells and in mouse fibroblasts expressing the human protease activated receptor (PAR)1, PAR2 or PAR4. HDM extract, Der p 1 and Der p 5 dose-dependently increased the production of IL-6 and IL-8. Added simultaneously, Der p 1 and Der p 5 further increased the production of IL-6 and IL-8. The action of Der p 1 was blocked by cysteine-protease inhibitors, while that of Der p 5 couldn't be blocked by either serine- or cysteine protease inhibitors. Der p 5 only induced cell shrinking, whereas HDM extract and Der p1 also induced cell desquamation. Der p 2 had no effect on A549 cells. Der p 1's protease activity causes desquamation and induced the release of IL6 and IL-8 by a mechanism independent of Ca^2+ ^mobilisation and PAR activation. Der p 5 exerts a protease-independent activation of A549 that involves Ca^2+ ^mobilisation and also leads to the production of these cytokines. Together, our data indicate that allergens present in HDM extracts can trigger protease-dependent and protease-independent signalling pathways in A549 cells.

## Background

House dust mite (*Dermatophagoides pteronissinus*) extracts contain allergens with potent sensitising capacities in atopic subjects. The sensitisation to HDM allergens is not only caused by exposure to allergenic compounds of the HDM but also by compounds that facilitate the access of allergens to cells of the immune system. Proteases produced by house dust mites (HDM) and fungi, or proteases present in pollen are able to decrease the barrier function of the epithelial cell layer. The proteases may disrupt the tight-junctions between epithelial cell and lead to the complete desquamation of the epithelial cell layer, hence facilitating the passage of allergens across the epithelial surface [1-3] Extracts of *Dermatophagoides pteronissinus *and *Lepidoglyphus destructor *have been shown to cause epithelial cell desquamation in a protease-dependent way. The result of the desquamation may be that allergenic compounds penetrate deep into the airway wall.

Airway-derived epithelial cells have been shown to increase the release of proinflammatory cytokines, such as interleukin (IL)-6 and IL-8, in response to proteases present in HDM-, pollen- and fungal extracts [4-7]. The release of cytokines may be mediated by protease activated receptors (PAR) that have been found on these cells [8,9]. Definitive proof for a PAR-mediated mechanism of these observations is hampered by the lack of specific PAR antagonists, but the use of human PAR expressed mouse fibroblast may elucidate whether a PAR is involved in the protease-dependent cytokine production [5]. In addition to protease-mediated mechanisms, a protease-independent activation of epithelial cells has been observed in studies with HDM extracts [4]. The latter observation suggested a possible interaction of airway epithelial cells with non-protease compounds of HDM extracts.

HDM extracts contain many proteins of known and unknown character, including Der p 1, Der p 2 and Der p 5. Der p1 has been shown to have cysteine protease activity [10-12] that may cause the observed epithelial cell desquamation, release of cytokines and facilitate transport of allergens across cultured epithelial cell layers [2,3,7,13]. Der p 2 and Der p 5 lack a clear-cut protease activity, but are major IgE binding proteins [14] and of unknown biological function [15]. In the present study we further elucidated the mechanism by which HDM extracts, a purified major allergen Der p 1, and three recombinant major HDM allergens (recDer p 1, recDer p 2, and recDer p 5) affect the biochemical properties of airway derived epithelial cells. We assessed how these compounds changed A549 cell morphology, whether they induced cell desquamation and their capacity to induce cytokine production. The mobilization of intracellular Ca^2+ ^and the involvement of protease activated receptors was analysed using mouse fibroblasts expressing human PAR1, PAR2 or PAR4.

## Methods

### House dust mite extract and (recombinant) allergens

Standardized lyophilized extracts of the house dust mite (*D. pteronissinus*) was a gift of Dr. Nico Niemeyer (ALK-Benelux, The Netherlands). Affinity chromatography purified natural Der p1 and the recombinant allergens (Der p 1 [16,17], Der p 2 [18], and processed Der p 5 [19]) were a generous gift of Dr. Martin D. Chapman (Indoor Biotechnologies Ltd, Cardiff, UK). Total protease (using casein as a substrate), elastase (using *N*-succinyl-alanyl-alanyl-prolyl-leucine *p*-nitro-anilide as a substrate) and gelatinase (using gelatin-orange as a substrate) activities of the mite extract were quantified as previously described [4].

### Epithelial cell lines and cell activation

A549 cells, a human alveolar type II epithelium-like cell line, were obtained from American Type Culture Collection (Rockville, MD). The epithelial cells were cultured in sterile 24-well culture dishes (Costar) in RPMI 1640 containing 5% heat-inactivated foetal calf serum complemented with 0.05% gentamycine to 90% confluency, as described previously [5]. Before incubation with the HDM extract or components, the cell cultures were incubated with serum-free medium during 24 hours (37°C). Stimulation of A 549 cells was performed with various concentrations of HDM or compounds there of (Der p1, Der p2 and Der p5) in serum-free medium complemented with LPS inhibitor colistin (10 μg/ml) at 37°C, 5% CO_2_. In order to have fully active purified Der p1 was reduced by incubating it with 0.5 mmol glutathione for 5 minutes before it was applied to the cell cultures. Chymostatin (10 μg/ml, Sigma) was used as non-specific protease inhibitor; Phenylmethylsulphonyl fluoride (PMSF, 0.25 mM, Sigma) was used as specific serine protease inhibitor; Trans-epoxysuccinyl L-leucylamido (4 guanidine) butane [E-64 (10 μM, Sigma)] was used as a specific cysteine protease inhibitor. Prior to addition, the protease inhibitors were incubated for 15 minutes (37°C) with HDM, Der p 1 and Der p 5 containing medium. Heat-treatment of media containing HDM extract, Der p 1 and Der p 5 was done at 65°C for 30 minutes. After 24 hours of incubation with the HDM components, supernatant was collected and stored at -20°C. Cytokine production was quantified using commercially available ELISA-kits for IL-6 (detection limit 1–3 pg/ml; Sanguin, Amsterdam, The Netherlands) and IL-8 (detection limit 4–8 pg/ml; Sanguin). Cell morphology was assessed by light microscope and quantified on an arbitrary scale (no effect = same morphology as non-treated cell; shrinking = visual changes in morphology predominated by cell shrinking and partial cell desquamation (≤10%) but no floating cells; desquamation = >10% cells have detached and are floating around) [5]. Cell viability was quantified using the trypan blue exclusion method. The presence of PAR receptors on A549 epithelial cells was checked by incubating the cells for 24 hrs with increasing concentrations of the PAR-1 and PAR-2 agonists. PAR1 (*NH2*-S-F-L-L-R-N-*C*) and PAR-2 agonist (*NH*_2_-S-L-I-G-K-V-*C*) were obtained from Eurosequence (Groningen, The Netherlands). The retrograde analogues of PAR-1 and PAR-2 were used to show specificity of the PAR-1 and PAR-2 agonists.

### Ca^2+ ^studies

Intracellular Ca^2+ ^measurements were performed on mouse fibroblasts expressing human PAR1, PAR2 or PAR4 (kindly provided by Dr Patricia Andrade-Gordon) and on A549 cells as previously described [20]. A549 cells were detached with protease-free buffer (CDS, Sigma) and resuspended in Hanks' solution at a concentration of 10^7 ^cells/ml. The cells were loaded with 2 mM indo-1/AM for 30 min at room temperature in the dark. Under these conditions, compartmentalization of the dye was minimal as judged from the ratio of fluorescence signals obtained after selective permeabilization of the plasma membrane (10 mM β-escin) and full permeabilization of the cells (1% Triton X-100). Then the cells were washed twice by centrifugation and their fluorescence was measured in an Aminco-Bowman spectrophotometer, using 10^6 ^cells/ml. Measurements were performed at 22°C, with a single excitation wavelength (349 nm) and a dual emission wavelength (410 and 490 nm) at a frequency of 1 Hz. Thapsigargin responses were measured at the plateau phase, which represents capacitative Ca^2+ ^influx. In Ca^2+^-free conditions, this plateau was not reached (see also [20]).

### Data analysis

All experiments were performed at least six times. Statistical analysis was performed with the student t-test. p values ≤ 0.05 were considered significant.

## Results

### House dust mite extract and Der p 1 induce morphological changes and cell desquamation in A549 cultures

As summarized in table I, both HDM extract and purified natural Der p 1 dose-dependently induced morphological changes. Low concentrations of these compounds were associated by cell shrinking, whereas higher concentrations lead to total cell desquamation of confluent A549 cell layers without affecting cell viability (all ≥97%). Both shrinking and desquamation were reversed by the protease inhibitors chymostatin (non-specific) and antipain (serine-proteinase specific inhibitor). Recombinant Der p 5 only caused shrinking of the A549 cells at the highest concentration (100 μg/ml), and did not induce desquamation of the cells. Recombinant Der p 2 neither affected cell morphology nor induced desquamation.

**Table 1:** Effects on cell shrinking and desquamation of house dust mite (HDM) extract and three recombinant allergens: Der p1, Der p2 and Der p5 (in μg/ml).

	No Effect	Shrinking	Desquamation
Der p1	0 – 1	10	100*
Der p5	0 – 10	100	NA
Der p2	1 – 100	NA	NA
HDM extract	0 – 1	2 – 10	50 – 400*

* = viability of cells ≥97%; NA = not applicable.

### HDM extract, natural and recombinant Der p 1, and Der p 5 induce cytokine release by A549 cells

**Figure 1:**
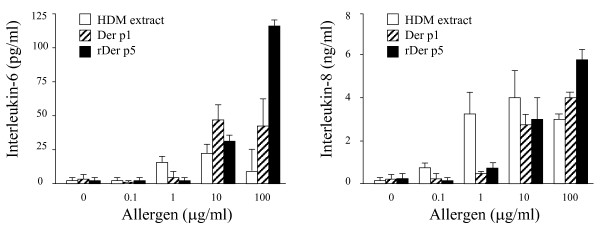
Dose-response of crude house-dust-mite (HDM) extract, natural purified Der p1 and recombinant (r)Der p5 of absolute levels of interleukin (IL)-6 (left panel) and IL-8 (right panel) protein. A549 cells were incubated during 24 hours in the absence and presence of increasing concentrations (indicated in μg/ml) of HDM extract, Der p1 and recombinant (r)Der p5. IL-6 protein levels are expressed as pg/ml; IL-8 protein levels are expressed as ng/ml.

**Figure 2:**
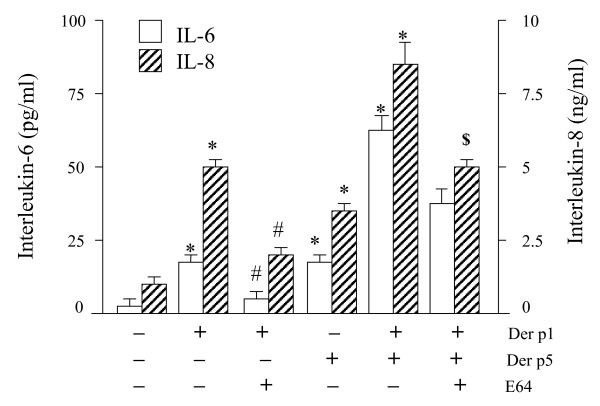
Effects of the cysteine protease inhibitor E64 (10 μM) on IL-6 (open bars) and IL-8 (hatched bars) protein secretion. A549 cells were stimulated during 24 hours with an optimal concentration of recombinant allergens: Der p 1 (20 μg/ml), Der p 5 (20 μg/ml) or a combination of Der p1 plus Der p 5. IL-6 protein levels are expressed as pg/ml, whereas IL-8 protein levels are expressed as ng/ml. * p < 0.05, significantly enhanced expression compared to negative control (medium); # p < 0.05, significantly diminished expression compared to Der p 1-induced levels; ^$ ^p < 0.05, significantly diminished expression compared to Der p1 plus Der p5-induced levels.

As demonstrated in figure 1, HDM extract, purified natural Der p 1 and recombinant Der p 5 induced a dose-dependent and significant increase of both IL-6 (n = 5, p < 0.05) and IL-8 proteins (n = 5, p < 0.05). The maximum level of cytokine production was achieved with 10 μg/ml HDM extract, while higherconcentrations reduced cytokine levels. Purified natural Der p 1 showed a maximal production of IL-6 (n = 6, p < 0.05) and IL-8 (n = 6, p < 0.05) at high concentrations (≥10 μg/ml). Reduction of natural Der p 1 with glutathione further increased the production of IL-6 and IL-8 (approximately 2-fold; data not shown), whereas the cytokine-inducing capacity of recombinant Der p 1 was not affected after glutathione treatment, indication that recombinant Der p 1 was already in its most active (reduced) form (data not shown). The recombinant Der p5 was the most potent inductor of cytokine production in A549 cells, and caused a dose-dependent and significant increase of both IL-6 (n = 6, p < 0.05) and IL-8 (n = 6, p < 0.05) with a maximal production at 100 μg/ml. In contrast, recombinant Der p 2 did not affect cytokine production, at all (data not shown). As shown in figure 2, the simultaneous addition of Der p 1 (20 μg/ml) plus Der p5 (20 μg/ml) further increased the production ofIL-6 (3-fold) and IL-8 (2-fold).

### Der p 1- and Der p 5-induced cytokine release are protease-dependent and protease-independent, respectively

**Figure 3:**
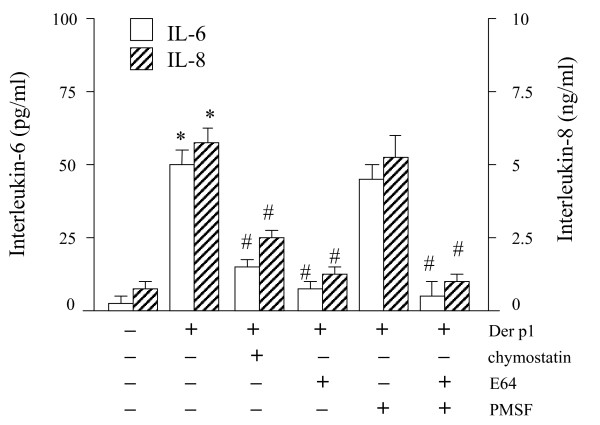
Effects of the several protease inhibitors on IL-6 and IL-8 protein secretion by A549 cells. Cells were stimulated during 24 hours with an optimal concentration of recombinant (r)Der p 1 (20 μg/ml), in absence and presence of optimal inhibitory concentrations of chymostatin (50 μg/ml; serine-protease inhibitor), E64 (10 μM; cysteine-protease inhibitor), or PMSF (0.25 mM; serine-protease inhibitor), or a combination of E64 plus PMSF. IL-6 protein levels are expressed as pg/ml, whereas IL-8 protein levels are expressed as ng/ml. * p < 0.05, significantly enhanced expression compared to unstimulated cells (medium); # p < 0.05 significantly diminished expression compared to rDer p 1.

Next, we determined the effects of heat treatment and protease inhibitors on allergen-induced production of IL-6 and IL-8. Heat treatment completely blocked natural Der p 1-induced cytokine release, while it only partially reduced the effect of recombinant Der p5 (IL-6 minus 28%, IL-8 minus 42%). As shown in figure 3, IL-6 and IL-8 production induced by the purified Der p 1 was completely inhibited by the cysteine-protease inhibitor E-64 (n = 5, p < 0.05). Chymostatin partially reduced purified Der p 1-induced cytokine production. In the presence of E-64, the higher levels of IL-6 and IL8 induced with a Der p 1 plus Der p5-induced IL-6 and IL-8 levels were diminished and comparable with levels induced by Der p 5 alone (figure 2).

### PAR2 agonist (*NH*_2_-S-L-I-G-K-V-C) increased cytokine production in A549 cells

**Figure 4:**
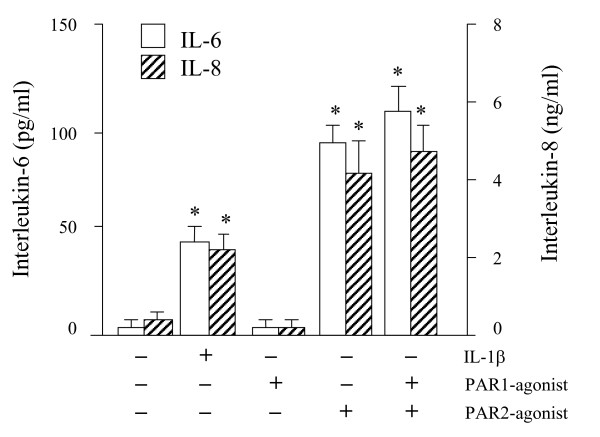
Effects of agonists for protease activated receptor (PAR)1 and PAR2. The agonists used for PAR-1 and PAR-2 are *NH2*-S-F-L-L-R-N-*C *and *NH*_2_*-S-L-I-G-K-V-C*, respectively. In the experiment shown the concentration of both PAR1 and PAR2 was 0.5 mM. IL-1β is shown as a positive control. * p < 0.05 compared to unstimulated cells (medium).

Because epithelial cells express protease-activated receptors (PAR), we examined whether a PAR-mediated mechanism is involved in HDM and Der p 1 induced cytokine production. PAR1 or PAR2 agonists were added to A549 cultures. As shown in figure 4, only the PAR2 agonist induced a dose-dependent increase of IL-6 and IL-8 protein and reached a maximal production at 5.10^-4 ^M, indicating the functional presence PAR2 receptors on A549 epithelial cells. Neither the retrograde analogue of PAR2 nor the retrograde analogue of PAR1 did affect IL-6 or IL-8 production, indicating the specificity of the PAR2 agonist.

### Der p 5 mobilises intracellular free Ca^2+ ^by a PAR-independent mechanism

**Figure 5:**
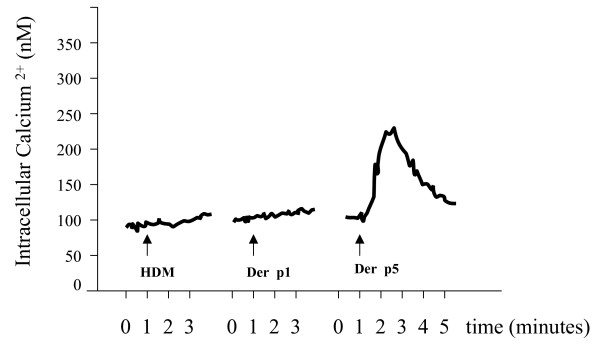
[Ca2+]_i _measurements in 10^6 ^A549 cells. 5 × 10^7 ^A549 cells were loaded with 2 μM indo-1/AM (see Materials and Methods), followed by washing and dual-wavelength measurement of fluorescence, using 10^6 ^cells/ml per measurement. [Ca^2+^]_i _was calculated as described. The three traces presented are representative for three independent experiments and show the effects of House dust mite extract (HDM), recombinant Der p 1 and recombinant Der p 5 on intracellular Ca^2+ ^homeostasis. Arrows indicate when the compounds were added.

The data obtained with the former experiments suggested that a functional PAR2 is expressed on A549 cells. It has been demonstrated that PAR activation leads to the mobilization of intracellular free [Ca^2+^], and, to further elucidate the underlying mechanism triggered by HDM, Der p 1 and Der p 5, we measured intracellular Ca^2+ ^levels in A549 cells treated in absence and presence of these compounds. As shown in figure 5, only Der p 5 was able to significantly mobilise [Ca^2+^]_i_. Finally, we assessed whether cytokine production induced by HDM, Der p 1 and Der p 5 is mediated via the activation of protease activated receptors (PAR). To this end we used mouse fibroblasts expressing the human PAR1, PAR2 or PAR4 and [Ca2+]_i _was measured. As expected, the PAR agonists trypsine (specific for PAR2) and thrombine (for PAR1 and PAR4) dose-dependently induce the mobilisation of [Ca2+]_i_, demonstrating the functional presence of the human protease activated receptors on the mouse fibroblast. In contrast, concentrations of HDM, Der p 1 and Der p 5 (10–20 μg/ml) that induced cytokine release in our previous experiments did not affect [Ca2+]_i _in these cells (data not shown).

## Discussion

Epithelial cells are important participants in the innate recognition of foreign substances. Aside from their mechanical barrier function, epithelial cells may also express surface receptors that are able to recognise components released from house dust mites. Here we show that airway epithelial cells interact with protease- and non-protease components from house-dust mites resulting in IL-6 and IL-8 release. In contrast to the purified and recombinant allergens, HDM extracts reached a maximum of activity (around 10 μg/ml), which is followed by a decline at higher concentrations. This bell-shaped dose-response profile that has also been observed for fungal extracts [5] suggests the presence of several activating components in the HDM extract, including cysteine- and serine proteases and Der p 5, that synergistically interact with the A549 cells. At very high concentrations the cytokine production was abrogated through an unknown mechanism, but coincided with total epithelial cell desquamation. Der p 1 has been shown to diminish the epithelial integrity through the destruction of the junctional proteins, in particular occludin and ZO-1 [2,21]. Der p 1-mediated break-down of these molecules may therefore explain the observed cell shrinking and desquamation in our studies.

In the present study we observed that the cysteine protease inhibitor E-64 blocked cell shrinking and desquamation, as well as the release of IL-6 and IL-8. The less specific protease inhibitor chymostatin also inhibited cell shrinking and desquamation as well as the production of cytokines. The latter finding is in agreement with observations that Der p1 has been shown to contain both cysteine and serine activity [12]. This dual cysteine-serine proteinase activity of Der p 1 has been a matter of debate, since the serine proteinase inhibitor 4-(2-aminoethyl)-benzenesulphonyl fluoride hydrochloride (AEBSF) did not affect Der p1 induced changes in permeability of epithelial monolayers [21]. In our present study the serine protease inhibitor reversed the Der p 1-induced effects in the presence of glutathione and suggested that Der p 1 has to be in its reduced state to provide functional serine-protease activity. The structurally very similar (recombinant) Der f 1 is strongly inhibited by the cysteine protease inhibitor E64 [22] and suggests that access to the active site of Der p 1 is hampered through steric hindrance and/or electrostatic interaction of substrates, thus preventing sufficient access to the enzymatic cleft. Recent three-dimensional space-filling studies of the Der p 1 molecule indicate that Der p 1 is not a serine protease, however [23]. Alternatively, the observed discrepancy between different studies might be due to the purity of the extracts used.

Our observation that Der p 1 activated the A549 epithelial cells to produce cytokines is consistent with the observation that proteases from house dust mites and fungal origin are able to activate NF-κB, a transcription factor critical for the production of IL-6 and IL-8 by epithelial cells [6]. Studies of protease-induced signalling, especially in platelets, endothelial cells and keratinocytes, have shown an abundance of G-coupled signalling pathways that are triggered upon cleavage of Protease-activated receptors (PARs) [24] PARs are also present on epithelial cells [8,9], including A549 cells [25,26], and the effect of Der p 1 may thus be mediated through cleavage of PARs, as has been reported for Der p 3 and Der p 9 [26]. Some conflicting reports have emerged in the literature regarding Der p 1 and PAR activation [27-29]. We demonstrated that a functional PAR2 is present on A549 cells by the specific PAR2 agonists, which induced the production of IL-6 and IL-8 in our studies. However, the mouse fibroblasts expressing the human PAR1, PAR2 or PAR4 demonstrated that the HDM extract, Der p 1 and Der p 5 did not affect intracellular calcium mobilization in these cells, and would rather argue against a PAR-mediated mechanism. That Der p 1 activates the release of cytokines from A549 cells in a PAR-independent manner is in accord with a recent study, showing that Der p 3 but not Der p 1 activated the PAR2 signalling cascade, hence inducing IL-8 [28]. An explanation for the conflicting reports would be contaminations of purified Der p 1 with Der p 3 as has also been suggested by Takai et al [29], and suggests that the Der p 1 used in our present studies is of high quality. To completely exclude a PAR2-mediated mechanism would require specific PAR2 antagonists, but those are currently not available.

The recombinant Der p 5 also induced the secretion of IL-6 and IL-8, and to an even higher extent than Der p 1. This effect of Der p 5 was dose-dependent, could not be blocked by protease inhibitors, and was specific, since recombinant Der p 2, another major HDM allergen, did not have any effect on the production of these cytokines. The combination of both Der p 1 and Der p 5 had an additive effect on IL-8 production and a synergistic effect on IL-6 production, demonstrating that Der p 5 activates a distinctly different intracellular signalling pathway than Der p 1. Der p 5 is of unknown biological functional [17], and the signalling pathways triggered by the Der p 5 have not been studies thus far. Here we showed that at least a calcium-dependent pathway might be activated by recombinant Der p 5. It may be hypothesised that receptors from innate recognition system, e.g. the Toll-like receptors, may be involved. If so, the synergistic interaction may be expected at the level of the activation of NF-κB [30,31] The HDM extract itself did not increase intracellular calcium levels, probably because the concentrations of Der p 5 and/or Der p3 in the HDM extract are insufficient to elicit this response.

In accordance with previous findings, we showed that the HDM-derived protease Der p 1 caused both damage and activation of airway epithelial cells. Damage to epithelial cells may facilitate the passage of allergens over the mucosal membrane, whereas an increased release of cytokines may induce an inflammatory response in the airway tissue. Whether the synergistic effect of Der p 1 plus Der p 5 causes results in the allergen to deeper penetrate into the airway wall, and enhances the immune response remains to be elucidated, but our observations may certainly contribute to non-allergic inflammatory responses in the airways.

## Conclusion

Allergens present in HDM extracts activate airway-derived epithelial cells in at least two ways: protease-dependent and protease-independent. Protease-dependent activation results in morphological changes, cell-desquamation and production of proinflammatory cytokines. Protease-independent activation further boosts production of proinflammatory cytokines, without affecting cell morphology. These two mechanisms may act synergistically, aggravating the ongoing inflammatory response observed in asthmatic airways. If we learn how to counteract these unexpected biological activities of allergens we might be able to develop novel treatments for atopic asthma patients.

## Abbreviations

HDM = house dust mite

Der p = dermatophagoides pteronissinus

PAR = proteinase activated receptor

IL = Interleukin

## Competing interests

The author(s) declare that they have no competing interests.

## Authors' contributions

HFK: ideas, study design and writing

MT: writing

JABT: ideas and laboratory work

PB: ideas, study design, laboratory work and writing

## References

[B1] Wan H, Winton HL, Soeller C, Tovey ER, Gruenert DC, Thompson PJ, Stewart GA, Taylor GW, Garrod DR, Cannell MB, Robinson C (1999). Der p 1 facilitates transepithelial allergen delivery by disruption of tight junctions. J Clin Invest.

[B2] Wan H, Winton HL, Soeller C, Gruenert DC, Thompson PJ, Cannell MB, Stewart GA, Garrod DR, Robinson C (2000). Quantitative structural and biochemical analyses of tight junction dynamics following exposure of epithelial cells to house dust mite allergen der p 1. Clin Exp Allergy.

[B3] Winton HL, Wan H, Cannell MB, Gruenert DC, Thompson PJ, Garrod DR, Stewart GA, Robinson C (1998). Cell lines of pulmonary and non-pulmonary origin as tools to study the effects of house dust mite proteinases on the regulation of epithelial permeability. Clin Exp Allergy.

[B4] Tomee JF, van Weissenbruch R, de Monchy JG, Kauffman HF (1998). Interactions between inhalant allergen extracts and airway epithelial cells: effect on cytokine production and cell detachment. J Allergy Clin Immunol.

[B5] Kauffman HF, Tomee JF, Van De Riet MA, Timmerman AJ, Borger P (2000). Protease-dependent activation of epithelial cells by fungal allergens leads to morphologic changes and cytokine production. J Allergy Clin Immunol.

[B6] Borger P, Koëter GH, Timmerman JA, Vellenga E, Tomee JF, Kauffman HF (1999). Proteases from *Aspergillus fumigatus *induce interleukin (IL)-6 and IL-8 production in airway epithelial cell lines by transcriptional mechanisms. J Infect Dis.

[B7] King C, Brennan S, Thompson PJ, Stewart GA (1998). Dust mite proteolytic allergens induce cytokine release from cultured airway epithelium. J Immunol.

[B8] D'Andrea MR, Derian CK, Baker SM, Brunmark A, Ling P, Santulli RJ, Brass LF, Andrade-Gordon P (1998). Characterization of protease-activated receptor-2 immunoreactivity in normal human tissues. J Histochem Cytochem.

[B9] Cocks TM, Fong B, Chow JM, Anderson GP, Frauman AG, Goldie RG Henry PJ, Carr MJ, Hamilton JR, Moffatt JD (1999). A protective role for protease-activated receptors in the airways. Nature.

[B10] Simpson RJ, Nice EC, Moritz RL, Stewart GA (1989). Structural studies on the allergen Der p1 from the house dust mite Dermatophagoides pteronyssinus: similarity with cysteine proteinases. Protein Seq Data Anal.

[B11] Chua KY, Stewart GA, Thomas WR, Simpson RJ, Dilworth RJ, Plozza TM, Turner KJ (1988). Sequence analysis of cDNA coding for a major house dust mite allergen, Der p 1. Homology with cysteine proteases. J Exp Med.

[B12] Hewitt CR, Horton H, Jones RM, Pritchard DI (1997). Heterogeneous proteolytic specificity and activity of the house dust mite proteinase allergen Der p I. Clin Exp Allergy.

[B13] Robinson C, Kalsheker NA, Srinivasan N, King CM, Garrot DR, Thompson PJ, Stewart GA (1997). On the potential significance of the enzymic activity of mite allergens to immunogenicity. Clues to structure and function by molecular characterization. Clin Exp Allergy.

[B14] Lynch NR, Thomas WR, Garcia NM, Di Prisco MC, Puccio FA, L'opez RI, Hazell LA, Shen HD, Lin KL, Chua KY (1997). Biological activity of recombinant Der p2, Der p5 and Der p7 allergens of the house-dust mite *Dermatophagoides pteronyssinus*. Int Arch Allergy Appl Immunol.

[B15] Thompson PJ (1998). Unique role of allergens and the epithelium in asthma. Clin Exp Allergy.

[B16] Recombinant Der p 1 product description. Indoor Biotechnologies Ltd. http://www.inbio.com/pdf_files/allergens/RPDP1.pdf.

[B17] Chapman MD, Smith AM, Vailes LD, Arruda LK, Dhanaraj V, Pomés A (2000). Recombinant allergens for diagnosis and therapy of allergic disease. J Allergy Clin Immunol.

[B18] Smith AM, Benjamin DC, Hozic N, Derewenda U, Smith WA, Thomas WR, Gafvelin G, Hage-Hamsten M van, Chapman MD (2001). The molecular basis of antigenic cross-reactivity between the group 2 mite allergens. J Allergy Clin Immunol.

[B19] Arruda LK, Vailes LD, Platts-Mills TA, Fernandez-Caldas E, Montealegre F, Lin KL, Chua KY, Rizzo MC, Naspitz CK, Chapman MD (1997). Sensitization to Blomia tropicalis in patients with asthma and identification of allergen Blo t 5. Am J Respir Crit Care Med.

[B20] Kok JW, Babia T, Filipeanu CM, Nelemans A, Egea G, Hoekstra D (1998). PDMPblocks brefeldin A-induced retrograde membrane transport from Golgi to ER: Evidence for involvement of calcium homeostasis and dissociation from sphingolipid metabolism. J Cell Biol.

[B21] Winton HL, Wan H, Cannell MB, Thompson PJ, Garrod DR, Stewart GA, Robinson C (1998). Class specific inhibition of house dust mite proteinases which cleave cell adhesion, induce cell death and which increase the permeability of lung epithelium. Br J Pharmacol.

[B22] Meno K, Thorsted PB, Ipsen H, Kristensen O, Larsen JN, Spangford MD, Gajhede M, Lund K (2005). The crystal structure of recombinant proDer p 1, a major house dust mite proteolytic allergen. J Immunol.

[B23] Hewitt CR, Brown AP, Hart BJ, Pritchard DI (1995). A major house dust miteallergen disrupts the immunoglobulin E network by selectively cleaving CD23: innate protection by antiproteases. J Exp Med.

[B24] Dery O, Bunnett NW (1999). Proteinase-activated receptors: a growing family of heptahelical receptors for thrombin, trypsin and tryptase. Biochem Soc Trans.

[B25] Dulon S, Cande C, Bunnett NW, Hollenberg MD, Chignard M, Pidard D (2003). Proteinase-activated receptor-2 and human lung epithelial cells: disarming by neutrophil serine proteinases. Am J Respir Cell Mol Biol.

[B26] Sun G, Stacey MA, Schmidt M, Mori L, Mattoli S (2001). Interaction of mite allergens Der p 3 and Der p 9 with protease-activated receptor-2 expressed by lung epithelial cells. J Immunol.

[B27] Asokananthan N, Graham PT, Stewart DJ, Bakker AJ, Eidne KA, Thompson PJ, Stewart GA (2002). House dust mite allergens induce proinflammatory cytokines from respiratory epithelial cells: the cysteine protease allergen, Der p 1, activates protease-activated receptor (PAR)-2 and inactivates PAR-1. J Immunol.

[B28] Adam E, Hansen KK, Astudillo Fernandez O, Coulon L, Bex F, Duhant X, Jaumotte E, Hollenberg MD, Jaquette A (2005). The house dust mite allergen DER P 1, unlike DER P 3, stimulates the expression of IL-8 in human airway epithelial cells via a proteinase-activated receptor -2 (PAR2) independent mechanism. J Biol Chem.

[B29] Takai T, Kato T, Sakata Y, Yasueda H, Izuhara K, Okumura K, Ogawa H (2005). Recombinant Der p 1 and Der f 1 exhibit cysteine protease activity but no serine protease activity. Biochem Biophys Res Commun.

[B30] Stacey MA, Sun G, Vassalli G, Marini M, Bellini A, Mattoli S (1997). The allergen Der p 1 induces NF-kB activation through interference with IkBα function in asthma bronchial epithelial cells. Biochem Biophys Res Commun.

[B31] Anderson KV (2000). Toll signaling pathways in the innate immune response. Curr Opin Immunol.

